# Ecological Insights, and Fin Fish Diversity in Carps Spawning Grounds: Case Studies from the Surma River and Tanguar Haor, Bangladesh

**DOI:** 10.1155/2024/7195596

**Published:** 2024-03-25

**Authors:** Kazi Rabeya Akther, Mohammad Amzad Hossain, Sohel Mian, Nirmal Chandra Roy

**Affiliations:** Laboratory of Aquatic Biodiversity and Ecophysiology, Department of Fish Biology and Genetics, Faculty of Fisheries, Sylhet Agricultural University, Sylhet 3100, Bangladesh

## Abstract

This study aims to provide a thorough ecological understanding of fin fish diversity within carp spawning grounds in the Surma River and Tanguar haor. Over two spawning seasons, this research investigates ecological factors impacting fin fish diversity and abundance in carp spawning grounds of the Surma River and Tanguar haor, emphasizing water quality, habitat features, spawn availability, and environmental influences. Fish spawn samples were collected with eight “Savar nets” at chosen sites and reared in a fiberglass tank at the mini hatchery for species identification. The representative spawn samples were examined under a microscope for preliminary species identification before rearing. The study found that both the Surma River and Tanguar haor offer ideal conditions for carp spawning due to factors such as water quality, natural food availability, habitat suitability, and favorable climatic conditions. The study identified 39 fish species under the 10 fish groups from both locations, with a higher percentage of carp species (31.42%) in the Surma River in 2021 compared to 2022 (22.50%). In Tanguar haor, the percentage of carp species was 7.55% and 6.50% in 2021 and 2022, respectively. The Surma River's ecological indices (2021-2022) indicated decreased diversity, likely due to environmental degradation, while Tanguar haor showed lower diversity possibly attributable to multiple environmental stressors. The dominant carp species, *Labeo calbasu*, *Labeo bata*, and *Labeo gonius*, were identified in both the Surma River and Tanguar haor. The spawning distribution varied among different locations, with some sites showing a presence of carp species, such as Hajipur (*T*_1_) in the Surma River and Alam Duar (*T*_2_) in Tanguar haor. The findings highlight the importance of hydrological and environmental parameters in shaping carp spawning habitat distribution and abundance, contributing to aquatic biodiversity conservation and resource management.

## 1. Introduction

Fish and fisheries play an important part in the economy and livelihoods of rural communities [1, 2]. The fisheries industry is one of the most remarkably productive and vibrant sector, contributing extensively to the country's socioeconomic development [3, 4]. After China and India, Bangladesh has the third-highest fish diversity in Asia, with over 800 distinct species [5, 6]. The freshwater fisheries assets are widely acknowledged for their remarkable abundance and variety, encompassing a minimum of 265 types of finfish [7, 8]. The Surma River and Tanguar haor are two essential aquatic ecosystems in Bangladesh that support a diversity of fish species and provide significant contributions to the country's fisheries industry [9–11]. The Surma River plays an important role in the fisheries of the Sylhet region. As a key drainage system in the region, the Surma River supports a diverse aquatic ecosystem, serving as an ideal habitat for numerous fish species [12, 13]. An ecological haven referred to as a sanctuary or safe place of unrivaled significance is the Tanguar haor, which is treated as the largest freshwater wetland in Bangladesh. This distinctive habitat contains a nexus of rivers, beels (oxbow lakes), and marshes to support diverse forms of an exquisite tapestry of species and is therefore designated as a Ramsar site [11, 14]. The fresh waters of the haor are home to a great array of flora and wildlife, including large carp that are essential to the food security and economic well-being of the surrounding areas.

Bangladesh's water bodies are home to the Indian major carp (IMC), encompassing four distinct species: Catla (*Catla catla*), Roho (*Labeo rohita*), Mrigel (*Cirrhinus mrigala*), and Kalibaush (*Labeo calbasu*) [15, 16]. These prominent carp varieties are highly regarded in the fisheries industry due to their commercial and nutritional value. Remarkably, major carp species contribute a substantial 26% to total fish production [17], effectively addressing nutritional requirements while bolstering revenue streams. Despite the ecological importance of major carp species and the vital role they play in the region's economy, knowledge of their natural spawning habitats and reproductive behavior is still inadequate. Based on the differences in spawning locations, spawning periods, and geographical dispersion, Bangladesh's major carp species are conventionally segmented into four stocks: (i) the stocks of the Brahmaputra-Jamuna, (ii) the upper Padma, (iii) the upper Meghna, and (iv) the Halda are listed in that order [18, 19]. There used to be natural carp spawning grounds in several rivers in the larger Sylhet area, including the Surma, Kushiara, and Monu Rivers [20, 21]. Aquaculture activities during the early 1960s and 1970s mostly involved raising natural carp hatchlings from the Jamuna, Padma, Boral, and old Brahmaputra rivers, as well as fertilized eggs during the monsoon from the Chittagong Halda River [22]. The natural carp seed population was 0.67% in 2012 and 0.39% in 2020, representing a 50% drop over the previous ten years [17, 23]. On the other hand, high-quality fish seed has been provided to the aquaculture sector by government and private hatcheries. Over time, fish seeds' quality has deteriorated, specifically in private hatcheries. Some of the factors influencing the poor quality and bringing the genetic variety of IMC (Indian Major Carp) natural populations into peril are genetic inbreeding, interspecific hybridization, and cross-breeding [24].

Owing to an interplay of diverse natural and human-induced influences, it is anticipated that spawning habitats may vanish or face extinction shortly [25]. As noted by Brander [26], climate change has demonstrated its impact not only directly on physiology, behavior, growth, reproduction, mortality, and distribution but also indirectly on the productivity, structure, and composition of aquatic ecosystems that serve as essential sources of sustenance and refuge for fish. In a prior study, it was also identified that fish have a close connection to both their biotic and abiotic environments. The life cycle, maturation, and growth of fish are strongly influenced by temperature, DO, pH, free CO_2_, alkalinity, and other salts. Small changes in such variables have an impact on fish growth, development, and maturity [27, 28]. The spawning sites used by the carp species are comparable, and they have similar environmental requirements [29]. Adult carp begin their spawning migration in early March due to rains, rising water temperatures, and increasing water flow from the Himalayas. Spawning starts in May with the arrival of the southwest monsoon rains and lasts until July [18, 30]. Very little research has been done on the spawning locations and spawning efficiency of major carp in Bangladesh. To enhance natural carp spawn production, it is crucial to investigate spawning sites in the Surma River and Tanguar haor. However, this study aims to provide a thorough ecological understanding of fin fish diversity within carp spawning grounds in the Surma River and Tanguar haor. The findings highlight the importance of hydrological and environmental parameters in shaping carp spawning habitat distribution and abundance, contributing to aquatic biodiversity conservation and resource management.

## 2. Materials and Methods

### 2.1. Study Area and Design

The investigation was carried out in the Surma River and Tanguar haor over the two-spawning periods from March to August 2021 and 2022. A large river in northeastern Bangladesh, the Surma River is well-known for having a remarkable impact on the hydrology and ecology of this area. This river's topology describes its structural features, including its course, tributaries, general spawning areas, spawning seasons, and geographic distribution.

**Figure 1:**
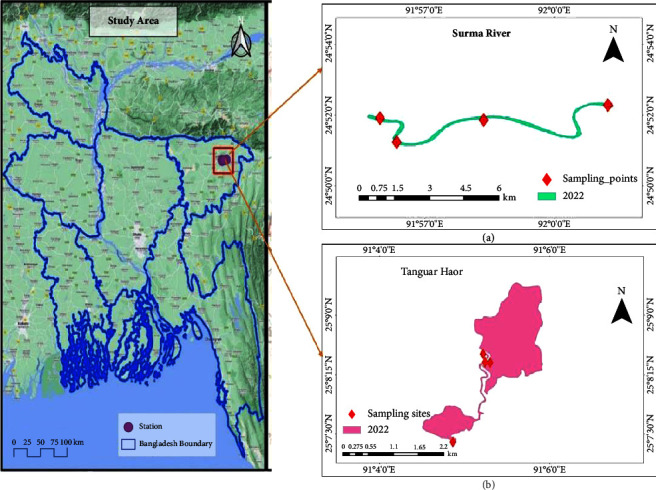
Map displaying the selected study area of the Surma River and Tanguar Haor, created using ArcGIS and Earth explorer.

Tanguar haor, located on the northern outskirts of Bangladesh at 25.1615°N and 91.0778°E, is one of the largest river basins, covering 9,727 ha (https://rsis.ramsar.org/ris/1031) in the Surma-Kushiyara river basins. It has a diverse ecosystem, including swamp forest land that plays a significant role in fish production and serves as a “Mother fishery” for the country [31]. Eight sampling transects were chosen from incentive areas along the Surma River and Tanguar haor with the help of the Upazila Fisheries Office, the fishing community, frequent visits, and previous research study [32] (Figure 1). These transects were divided into groups consisting of reference and treatment for the Savar net setting and sample collection (Table 1). Each station contains one spawning reference location that has been described in prior research.

### 2.2. Water Quality Measurement

Key parameters such as temperature, dissolved oxygen (DO), pH, turbidity, total dissolved solids (TDS), conductivity, and ammonia play pivotal roles in assessing the overall health of aquatic ecosystems. Temperature, DO, pH, and turbidity were measured using precision instruments including the Hack multimeter, PHC 101 pH probe, DO probe, and Sacchi disk. TDS and conductivity were measured using both the amber bottle and beaker method, as well as a multiparameter device featuring a CDC-401 probe. The concentration of ammonia, a crucial indicator of water quality, was assessed using the HACH HQ40d multiparameter analyzer. Daily water quality assessments were carried out using a range of instruments during the critical spawning period. This systematic approach ensures the thorough monitoring and management of aquatic environments, contributing to the preservation and sustainability of ecosystems.

### 2.3. Natural Food Abundance (Plankton Analysis)

The plankton samples were thoroughly collected from the study area during the last week of each month, precisely between 8 and 9.30 a.m. The collection process involved sieving 50 liters of water from the habitat, approximately 8–10 cm below the water's surface. This water was then transported through a 25 *μ*m mesh plankton net to capture the plankton. Subsequently, the collected plankton was concentrated to 25 ml. To preserve the samples for further analysis, a 5% formalin solution was used. The samples were carefully stored in a dark environment for one day to facilitate the precipitation process while minimizing the influence of sunlight, ensuring accurate measurements. The qualitative and quantitative analysis of the plankton was conducted using an electric microscope (Olympus Xcam-Alpha, Germany) to provide detailed insights into their characteristics and abundance. Each sample was stirred gently before examination under the microscope. The identification of plankton was performed using keys provided by [33] and APHA [34]. To count the plankton number per liter sample using the drop count method, the following formula was applied:

(1) $$\begin{array}{c} \text{Total plankton count per liter}=A\times \left(\frac{1}{L}\right)\times \left(\frac{n}{v}\right), \end{array}$$

where *A* = number of organisms per drop, *L* = volume of the original sample (50 liters); *n* = total volume of the concentrated sample (25 ml), *v* = volume of one drop (0.04 ml). In this study, n was 25 ml, L was 50 liters, and v was 0.04 ml (equivalent to 25 drops per 1 ml).

### 2.4. Meteorological Condition Analysis and Habitat Characteristics

The meteorological and environmental data related to precipitation and water level were collected from the data repository of the Power Data Access Viewer of NASA and https://www.hydrology.bwdb.gov.bd (retrieved on June 21, 2022). In addition, the habitat characteristics of the chosen study area were visually observed through regular visits to the site.

### 2.5. Fish Spawn Collection and Identification Process

**Figure 2:**
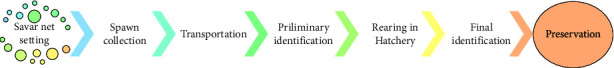
Flowchart detailing the collection and identification process of carp spawns from the Surma River and Tanguar Haor.

The fish spawn at the study sites was collected by using a Savar net. The fish spawn was sampled at six-hour intervals in each site employing a Savar net in the *hapa* setting. To determine the presence of carp spawn in the collected samples, a series of further steps were followed, which involved sample collection, transportation, rearing, and eventual identification in the laboratory (Figure 2). Subsequently, these samples were transported to the SAU's mini hatchery using oxygen bags for further examination.

The spawn collection process is outlined below:

#### 2.5.1. Savar Net Preparation and Setting

The traditional Indian major carp spawn fishing technique uses a “Savar net,” a fixed funnel-shaped net deployed mainly in the Ganges-Padma and Brhamaputra-Jamuna River systems. This net is small, anchored to shallow river shores, and equipped with a collection pocket. It catches microscopic fish eggs during the monsoon season. To prevent escape, the upper tail bag border is kept 4-5 cm above the water. In this research study, eight nets were placed strategically at sampling stations 30–50 meters apart in the Surma River and Tanguar haor. Professional fishermen collected fish spawn samples from each net monitored four times daily at six-hour intervals.

#### 2.5.2. Spawn Collection

In 2021, the Savar net on the Surma River captured 1.01 kg of fish spawn, leading to a 200-gram sample for analysis. By 2022, this amount had surged to 3.90 kg, prompting a larger 400-gram sample collection. In contrast, in 2021, only 0.40 kg of fish spawn was found in the Tanguar haor Savar net, leading to a 100-gram sample. By 2022, this had increased to 0.60 kg, with a 200-gram sample collected for analysis.

#### 2.5.3. Spawn Transportation to SAU Mini Hatchery, Rearing, Identification, and Preservation

Upon gathering the samples, they were promptly transported and placed in the raising tank at SAU's mini hatchery. After preliminary identification, the collected samples were cultivated for 30 days. During this period, the fish seeds were meticulously reared using appropriate management techniques, including feeding with egg yolk and subsequently floating powder feed from mega feed to facilitate nursing. After 30 days, the fish spawn was about 1.5 to 2.5 cm in size and easily identifiable. During the stocking of spawn in the raising tank, the ocular examination is used to identify fish species and finally preserve the fish.

### 2.6. Diversity Analysis

The Shannon–Wiener index (*H*′) the Margalef index (*d*), Pielou's index (*J*′) the Simpson index (*c*), Fisher's alpha evenness, and Berger–Parker dominance were used to analyze the species cluster's diversity [35–37].

  Shannon–Wiener diversity index (*H*′) : *H*'=SUM [*Pi* × log(Pi)]; Where, *H*′ = Shannon–Wiener index; *Pi*=*ni*/*N*; *ni* = no. of individuals of a species; *N* = total number of individuals
  Margalef species richness (*d*) : *d*=(*S* − 1)/ log(*N*)*;* where, *S* = total species*; N* = total individuals.
  Pielou's evenness index (*J*′) : *J*′=(*H*(*s*)/*H* (max)); where, *H*(*s*) = the Shannon–Wiener information function; *H*(max.) = the theoretical maximum value for *H*(*s*) if all species in the sample were equally abundant.
  Simpson dominance index (*c*):*C*=∑_*n*=1_^*s*^ni/*N*; Where, *ni* = number of individuals in the “each” species; *N* = total number of individuals; *S* = total number of species
  Fisher's alpha evenness: *S*=*a∗* ln (1+*n*/*a*) where *S* is the number of taxa, *n* is the number of individuals, and *a* is Fisher's alpha.
  Berger–Parker dominance: *d*=*N* max \*N* where *N*max is the number of individuals in the most abundant species, and N is the total number of individuals in the sample.

### 2.7. Data Processing and Analysis

Before undergoing statistical analysis, the obtained data was organized, processed, and compiled in Microsoft Excel (2010). Statistical analysis and interpretation of the raw data, using Kruskal–Wallis ANOVA, were conducted using SPSS v24. The Violin box Plot was performed in R-studio, PAST (version 4.03) was used for biodiversity indices, ArcGIS 10.8 and Google Earth Explorer were used for map formation [38]. The normality was tested by using Shapiro–Wilk, and the Kolmogorov–Smirnov Test (Supplementary table 1). As data did not exhibit a normal distribution pattern, the Kruskal–Wallis test was employed at *p* < 0.05 for comparing means (Supplementary table 2).

## 3. Results

### 3.1. Water Quality Environment

**Figure 3:**
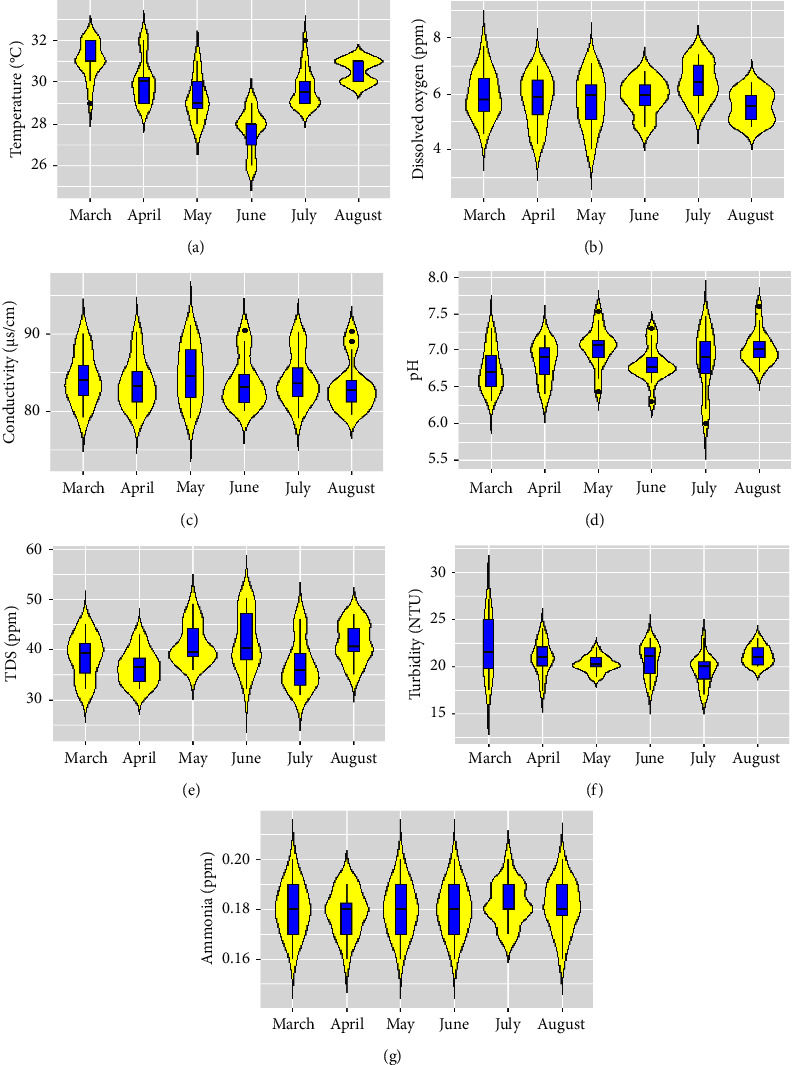
Display box plots illustrating the monthly variation of water quality parameters: (a) temperature, (b) dissolved O_2_, (c) conductivity, (d) pH, (e) TDS, (f) turbidity, and (g) ammonia.

Spawns were collected from the Surma River and Tanguar haor between July 10th and July 14th in 2021 and on June 2nd and 3rd in 2022. During this period, the average temperature of the selected study site was 28°C, where the maximum temperature (33°C) was found in March and the lowest temperature (28°C) was observed in June and July. The dissolved oxygen was found to be 5.9 to 7.4 ppm during spawning time, and the maximum value was observed at spawning time. The pH of the spawning period was 7.4, which was satisfactory for spawning (Table 2 and Figure 3). The TDS ranges from 37 to 39 ppm during the spawning period. Water turbidity is very important for the spawning of carp species, with observed values of 18–20 NTU during the spawning time. The ammonia level in the study area was ideal in both years (Table 2). The maximum value of the EC was found to be 89 *μ*S/cm, and the lowest was 80 µS/cm. During the spawn collection time, the EC was observed at 84–87 *μ*S/cm in both stations (Figure 3).

### 3.2. Natural Food Abundance

Carp species rely on plankton as a crucial food source for their growth, survival, and reproduction. Plankton serves as an indicator for assessing the productivity and fishery potential of different water bodies due to their importance in the fish food chain.

**Figure 4:**
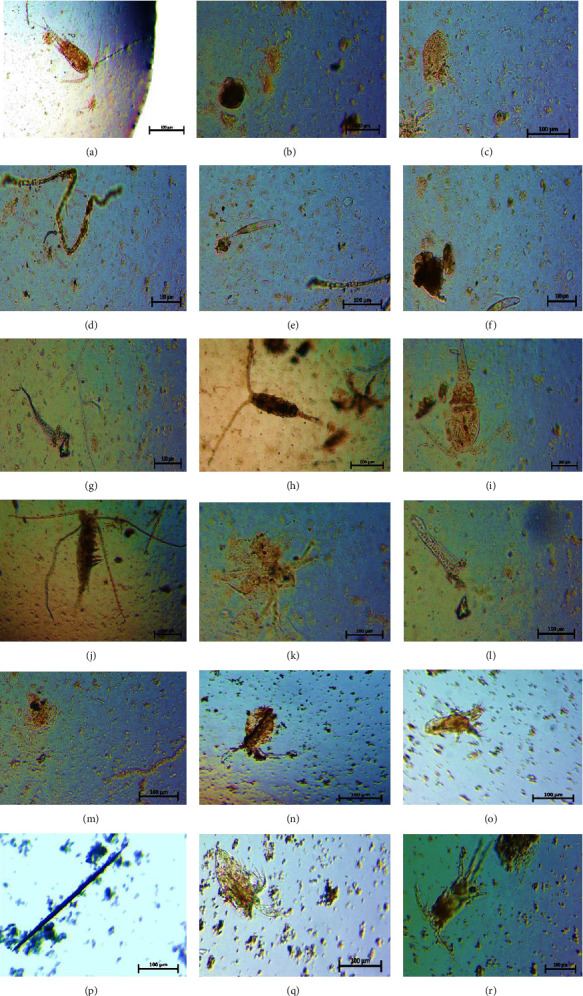
Illustrates the plankton community in Surma River and Tanguar Haor, featuring various species: (a) *Diaptomus* sp., (b) *Asplanchna* sp., (c) *Daphnia* sp., (d) *Spirulina* sp., (e) *Euglena* sp., (f) *Brachionus* sp., (g) *Ceratium* sp., (h–j) and (r) *Cyclopes* sp., (k) *Sid* sp., (l) *Ulothrix* sp., (m) *Nauplius*, (n) *Moina* sp., (o) *Diaphanosoma* sp., (p) *Synedra* sp., and (q) Rotifer.

A total of 37 planktonic taxa were found among them 23 taxa belonged to phytoplankton and 14 taxa were classified as zooplankton. Chlorophyceae (11 taxa), Bacillariophyceae (4 taxa), Cyanophyaceae (6 taxa), and Euglenophyceae (2 taxa) were further divisions of the phytoplankton group. Rotifer (4 taxa), Cladocera (5 taxa), Copepods (3 taxa), and Protozoans (2 taxa) were all included in the zooplankton group (Tables 3(a) and 3(b), and Figure 4).

**Figure 5:**
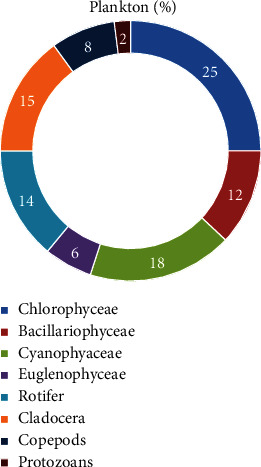
Percentage of planktonic groups in the Surma River and Tanguar Haor.

The findings recorded Chlorophyceae as most abundant, constituting 25% of the population, while protozoans were the least abundant at 2%. Notably, Rotifers, which are essential zooplankton for carp feed, were found at a significant abundance of 14%. Overall, the planktonic community's abundance demonstrated that the study area provided a rich natural food source for carp species during the spawning period (Figure 5).

### 3.3. Meteorological Condition During the Spawning Period

**Figure 6:**
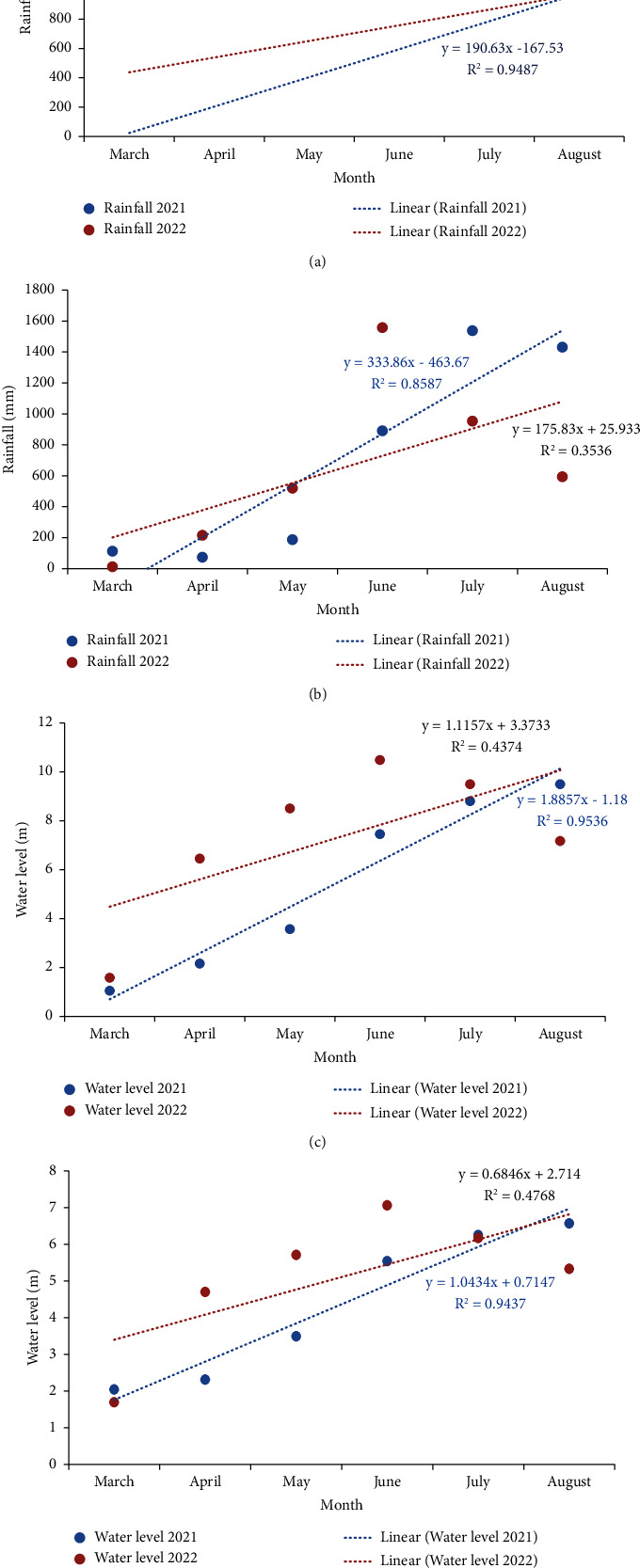
Linear regression of monthly rainfall (a, b) and water level (c, d) in the Surma River and Tanguar Haor, respectively.

The minimum rainfall and water level were noted in March, while the maximum values were observed in June and July. This pattern suggests a correlation between rainfall and water level fluctuations and the spawning success of carp species at the study site. These environmental factors were likely to play a significant role in influencing the conditions for successful carp spawning (Figure 6).

### 3.4. Habitat Characteristics for Carp Spawning

The Surma River exhibits distinct sections and habitats, featuring slower-moving segments with gravelly or sandy bottoms, alongside riverbanks adorned with vegetation and submerged objects such as boulders and tree roots. In contrast, the shallow, marshy regions of Tanguar Haor harbor submerged plants ideal for spawning, offering a conducive environment for safeguarding fry and attaching eggs of carp species.

### 3.5. Fish Spawn Status and Carp Spawning

**Figure 7:**
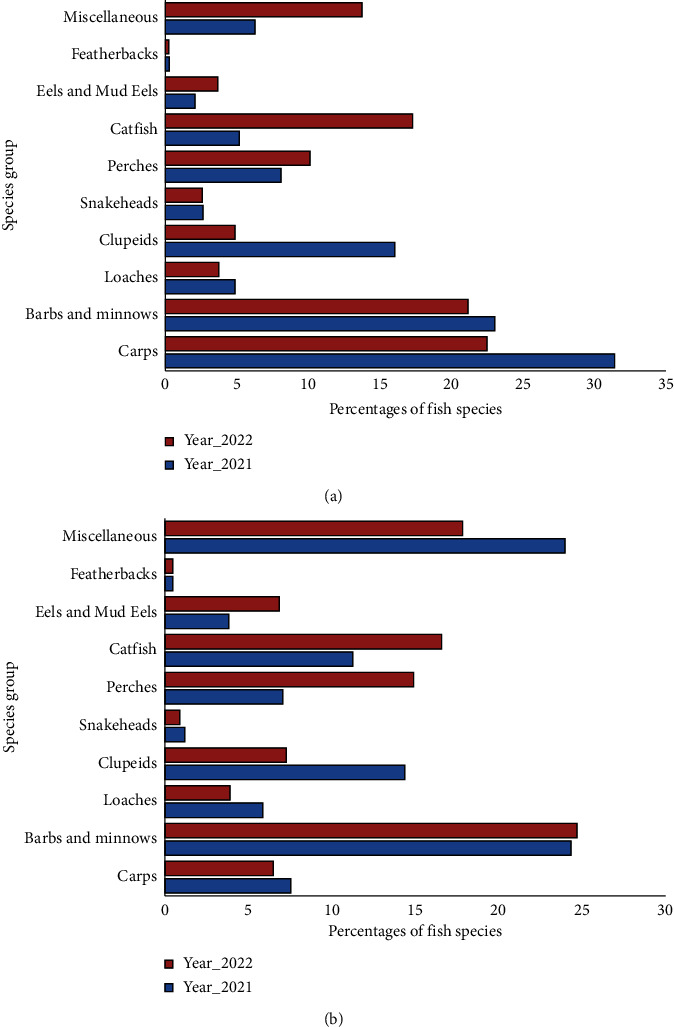
Fish species composition (%) in spawn samples from (a) Surma River and (b) Tanguar Haor.

After 30 days of cultivation in a small hatchery at SAU, the fish spawn reached a size of approximately 1.5–2.5 cm and was successfully identified. The study yielded a total of 10 groups of fish species from both the Surma River and Tanguar haor, comprising 39 different fish species (Figure 7). The analysis of the collected data showed that carp spawn had the highest percentage (31.42 and 22.50) among the fish species in the Surma River, while featherbacks had the lowest percentage (0.29 and 0.26). Conversely, in Tanguar haor, barbs and minnows had the highest percentage, and featherbacks had the lowest. Specifically, in the year 2021, the carp species accounted for 31.42% of the fish population in the Surma River and 22.5% in the year 2022. In Tanguar haor, the percentage of carp species was 7.55% in 2021 and decreased to 6.50% in 2022.

**Figure 8:**
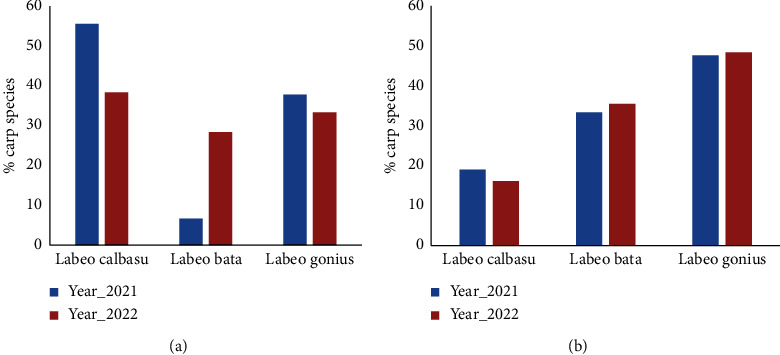
Carp species composition (%) in spawn samples from (a) Surma River and (b) Tanguar Haor.

**Figure 9:**
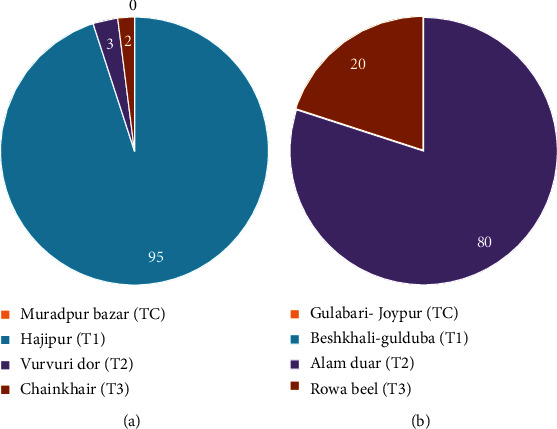
Compares the composition of carp species (%) in spawn samples from two stations (a) Surma River and (b) Tanguar Haor.

The findings indicated the presence of minor carp species, namely, *Labeo calbasu*, *Labeo bata*, and *Labeo gonius*, both in the Surma River and Tanguar haor. In the case of the Surma River, the carp species *Labeo calbasu* exhibited the highest percentage (55.55% in 2021 and 38.33% in 2022), while *Labeo bata* showed the lowest percentage (6.67% in 2021 and 28.33% in 2022). Conversely, in Tanguar haor, the carp species *Labeo gonius* displayed the highest percentage (47.63% in 2021 and 48.39% in 2022), whereas *Labeo calbasu* had the lowest percentage (19.04% in 2021 and 16.13% in 2022) (Figure 8). The study revealed that most of the carp spawn in the Surma River station, a significant portion, 95%, of the carp species was collected from Hajipur (*T*_1_), whereas the lowest numbers were observed in Vurvuri Dor (*T*_2_) and Chainkhair (*T*_3_). Moreover, no carp spawn was found in Muradpur Bazar site. In Tanguar haor was discovered in Alam Duar (*T*_2_) accounting for 80% of carp spawn, while the remaining 20% was found in Rowa Beel (*T*_3_). However, no carp spawn was found in Gulabari-Joypur and Beshkhali-Gulduba (*T*_1_) (Figure 9).

### 3.6. Spawn Fish Diversity

**Figure 10:**
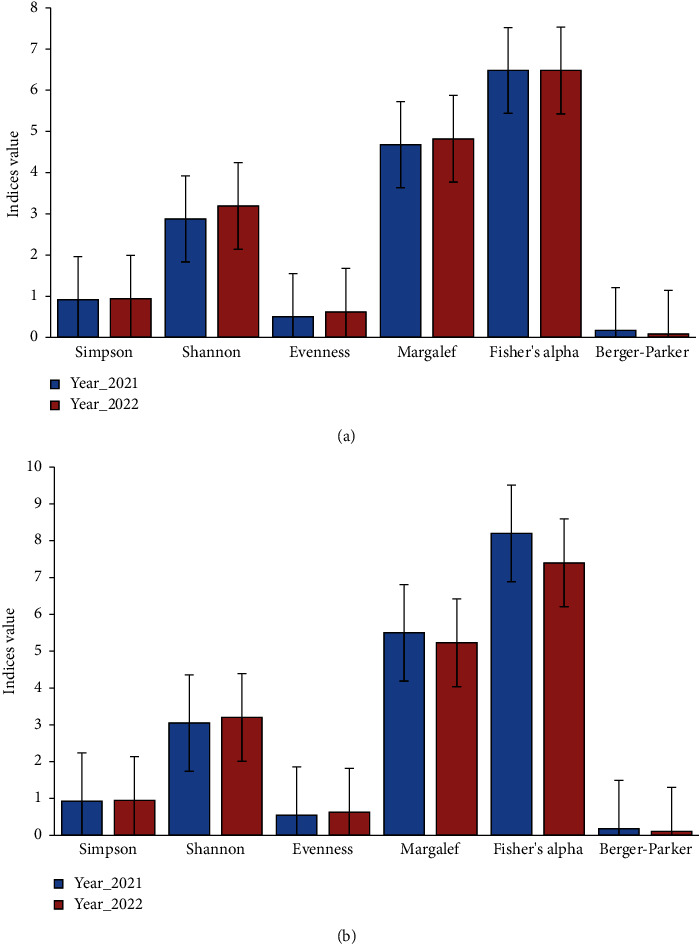
Fish diversity indices: (a) Surma River and (b) Tanguar Haor.

Figure 10 illustrates six fish diversity indices across two study locations. In this context, the observed differences in fish diversity indices between the Surma River and Tanguar haor over two years suggest contrasting ecological dynamics in these two study locations. During the study conducted in 2021 and 2022 in the Surma River, various diversity indices were assessed. Simpson's index exhibited values of 0.92 and 0.94, while Shannon's index showed figures of 2.879 and 3.192. Evenness values were recorded as 0.508 and 0.624, with Margalef's index at 4.68 and 4.82 and Fisher's alpha remaining constant at 6.48 for both years. The Berger–Parker indices displayed values of 0.17 and 0.09. Notably, the lower Simpson, Evenness, and Berger-Parker indices in the Surma River suggest a decline in diversity. Such a decrease could potentially be attributed to various factors, including habitat degradation, pollution, or overfishing. Conversely, the higher Shannon, Margalef, and Fisher's alpha indices in the same location suggest a comparatively healthier fish community, possibly due to better habitat quality or management practices.

During the study conducted in 2021 and 2022 in Tanguar Haor, several diversity indices were evaluated. Simpson's indices showed values of 0.93 and 0.95, while Shannon's indices were recorded as 3.05 and 3.2. Evenness values were 0.55 and 0.63, with Margalef's indices at 5.5 and 5.23. Fisher's Alpha exhibited values of 8.2 and 7.4, while Berger-Parker indices displayed figures of 0.18 and 0.11. The lower diversity indices observed in Tanguar haor compared to the Surma River suggest a less diverse fish population in this area. This phenomenon could be influenced by various factors, including habitat fragmentation, invasive species, or natural resource exploitation. However, further investigation is needed to understand the specific drivers behind these patterns and their implications for ecosystem health and management.

**Figure 11:**
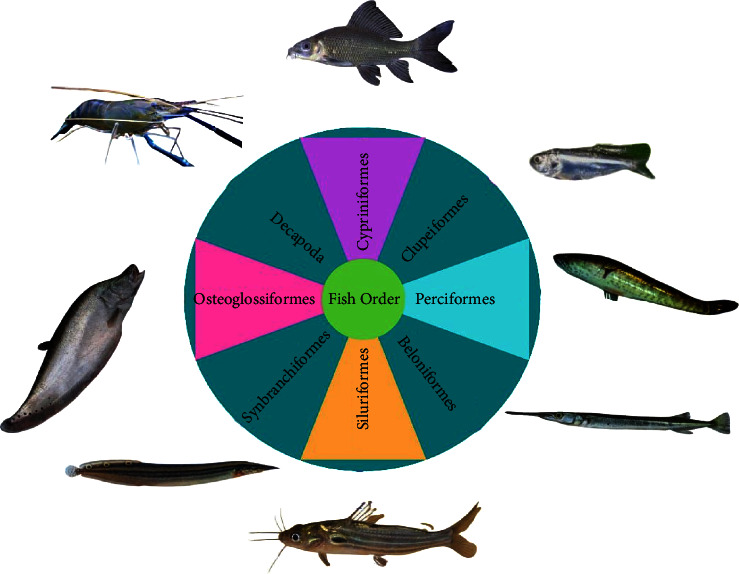
Order of freshwater fishes with representative species from the Surma River and Tanguar Haor.

Eight orders were identified, with Cypriniformes exhibiting the highest species diversity, primarily dominated by carp species (Table 4 and Figure 11). Upon collecting the fish sample, the Savar net contained various small indigenous species of fish (SIS), including Mola (*Amblypharyngodon mola*), deshi Puti (*Puntius* sp.), Darkina (*Esomus danricus*), Chela (*Chela cachius*), Tengra (*Mystus* sp.), and Baim (*Mastacembelus* sp.), among others (Table 4 and Figure 11). In comparison to carp species, the SIS displayed a significantly larger size. The SIS varied in size from 2.5 to 4.0 cm, depending on the specific type of fish.

## 4. Discussion

The successful carp spawning in the Surma River and Tanguar haor can be attributed to several key factors. The favorable water quality, availability of natural food, suitable habitat, and favorable climatic conditions provided an ideal environment for carp reproduction in both ecosystems. These findings emphasize the importance of maintaining and preserving these vital components of the aquatic environment to support healthy fish populations. In the past, many rivers in greater Sylhet, especially the Surma, Kushiara, and Monu rivers, served as the natural spawning grounds for various carp species [13, 22, 39]. The selection of the study area was made based on previous research studies and frequent visits. Scientists examined whether the parameters of temperature, turbidity, dissolved oxygen (DO), carbon dioxide (CO_2_), pH, chloride, nitrite, and acidity at the Hetimganj site adhered to the established standard limits for fisheries. The levels of total dissolved solids (TDS), alkalinity, and hardness were notably reduced in the rainy season [13, 40]. During the spawning time, the temperature, DO, pH, TDS, turbidity, and conductivity were 28°C, 7.4 ppm, 7.4, 34 ppm, 19.5 NTU, and 81 *μ*S/cm, respectively. According to the previous study, the accepted threshold for alkalinity is greater than 100 ppm, while transparency should measure 40 cm or below [41]. TDS has a standard limit of 165 ppm and hardness has a standard limit of 123 ppm [42, 43], while pH has a standard limit of 6.5 to 8.5 and DO has a standard limit of 5.0 ppm, as referenced by scientists [16, 44]. The observed trends in carp species abundance and distribution highlight the significant influence of environmental factors on spawning success. The higher percentage of carp species in the Surma River compared to Tanguar Haor indicates variations in habitat suitability and water quality between these two locations.

The study revealed that 23 taxa of phytoplankton were identified, distributed among four groups: Chlorophyceae (11 taxa), Bacillariophyceae (4 taxa), Cyanophyaceae (6 taxa), and Euglenophyceae (2 taxa). These findings were consistent with previous studies [45–48]. It was also noticed that a total of 14 taxa of zooplankton were identified, categorized as Rotifer (4 taxa), Cladocera (5 taxa), Copepods (3 taxa), and Protozoans (2 taxa). This finding is aligned with previous research [45] in the beels of the haor region in Bangladesh, where they identified 15 major taxa of zooplankton belonging to the Rotifer, Cladocera, and Copepod groups. Researchers [46] also conducted a study and identified 24 different taxa of zooplankton, including Rotifer, Cladocera, Copepod, and Ostracoda. However, this diverse planktonic community likely serves as a significant food source for carp, supporting their spawning success. The abundance and distribution of phytoplankton, such as Chlorophyceae, Bacillariophyceae, Cyanophyaceae, and Euglenophyceae, along with zooplankton taxa like Rotifer, Cladocera, Copepods, and Protozoans, may directly influence the availability of natural food for carp during spawning periods. Consequently, variations in planktonic community composition and abundance could affect the reproductive success of carp populations in these aquatic ecosystems.

The weather was beneficial for rainfall and temperatures during the spawning time. Unpredictable rainfall patterns from late February through the first week of March led to inadequate water levels in the rivers and beels of haors. However, this phenomenon rapidly triggers fish breeding. While some fish species manage to breed during these unfavorable rainfall conditions, insufficient water levels hinder the larval development of fish [47]. Furthermore, due to the irregularity and scarcity of rainfall, along with reduced water levels in interconnected rivers and canals, the broodfish struggle to reach the breeding grounds promptly [48]. As a result, the climatic fluctuations caused by erratic rainfall disrupt breeding activity and fish diversity. It was also denoted by the present study that there was a possession of optimal spawning habitat with slower river flow, vegetation, marshy land, and submerged substrate for carp. Shallow, slow-flowing waters with submerged vegetation or plant debris, sandy bottom, and stone were suitable for carp spawning (water.vic.gov.au).

The comprehensive investigations conducted during this study led to the identification of a diverse array of fish species. A total of 39 different fish species belonging to 10 groups were observed in both the Surma River and Tanguar haor. This biodiversity showcases the ecological richness of these aquatic ecosystems and highlights their significance in sustaining various fish species, including carp. The study also revealed interesting trends in the percentage of carp species over the two-year observation period. Notably, in the Surma River, the percentage of carp species was higher in 2021 (31.42%) compared to 2022 (22.5%). Similarly, in Tanguar haor, the percentage of carp species showed a decline from 7.55% in 2021 to 6.50% in 2022. The variations in carp species percentages over two years suggest potential shifts in fish populations in the Surma River and Tanguar haor. Various ecological factors, like habitat alterations, water quality fluctuations, and human activities, may influence these changes. Further investigation through long-term monitoring and ecological assessments is necessary to understand the underlying drivers and develop conservation strategies for sustaining fish populations in these ecosystems. In a study conducted near Sylhet Sadar, northeastern Bangladesh, the Surma River is known to harbor 51 fish species, categorized into 16 taxonomic groups [7, 10]. The dominant family was Cyprinidae, accounting for 36% of the species which was closely related to the present study. The absence of carp spawn at the Muradpur bazar site may be attributed to the absence of brood fish, likely due to the lack of deeper portions in this segment of the Surma River. Over the past two decades, 143 species of fish (137 native and 6 exotic) belonging to 35 families and 12 orders have been identified. Several studies have extensively documented the fish diversity within the Tanguar Haor area [49]. Roy et al. [50] reported that carp seed collection varied significantly between Hetimganj and Bhadeswar points, with 50 g and 3.105 kg of seeds assembled, respectively. Moreover, at Hetimganj point, the fishing intensity for carp spawn exhibited a range from 186.56 g/day/net during the new moon to 20 g/day/net during the full moon, while both Monumukh and Atgram points showed no detectable carp fish seeds. However, factors such as water quality, natural food availability, habitat suitability, and climatic conditions play pivotal roles in creating conducive environments for carp spawning. The decline in carp species diversity over the study period, particularly in the Surma River, underscores the impact of environmental degradation on spawning habitat quality. Similarly, the lower diversity observed in Tanguar haor suggests the presence of multiple stressors affecting the ecological balance of this wetland ecosystem.

The research identified the presence of three minor carp species, specifically *Labeo calbasu*, *Labeo bata*, and *Labeo gonius*, in both the Surma River and Tanguar haor. The distribution of carp spawning sites varied among different locations, with certain areas showing a substantial presence of carp, such as Hajipur (*T*_1_) in the Surma River and Alam Duar (*T*_2_) in Tanguar haor. The study determined that there may be possibilities for the natural spawning grounds of carp species. This indicates the presence of natural spawning sites for carp within the Surma River, as exemplified by the presence of carp fish seeds both in the Surma River and Tanguar haor. Furthermore, the variation in spawning distribution among different locations within the study sites emphasizes the importance of local habitat characteristics in shaping carp spawning behavior and abundance. Sites like Hajipur (*T*_1_) in the Surma River and Alam Duar (*T*_2_) in Tanguar haor emerge as potential hotspots for carp spawning, indicating the need for targeted conservation efforts in these areas.

Researchers found that in the upper Meghna in Bangladesh, there is virtually limited information on carp spawning sites and spawn collection points. There are no commercial carp spawn harvesting centers in the upper Meghna River basin, unlike in other river systems [32]. This stock's spawning could occur hundreds of miles upstream in India, or it could be so scarce that fry/spawn collectors are not interested. However, some authors claim that spawn collection locations can be found around the Surma River's headwaters in Manipur province, as well as in Tripura province in India [22, 51]. Recent studies have identified seven carp spawn collecting areas in the larger Sylhet basin based on fishermen's catch data provided by Paul [52]. These areas include the Juri River in Hakaluki haor near the Fenchugonj Bridge; the Kawani, Boroiya, and Baulai Rivers near Daulatpur and Milonpur in the Dharampasha Upazila; the Baulai River near Alamduarer bank in the Tahirpur Upazila; and the Dhanu river near Ranichapur. This study also suggests that potential spawning grounds for carp species could be located around the Surma River and Tanguar haor, where deeper water portions, locally referred to as “Dor,” are present.

## 5. Conclusions

The study offers a comprehensive understanding of the ecological dynamics and finfish diversity in the carp-spawning grounds of the Surma River and Tanguar haor in Bangladesh. Critical factors such as water quality, food availability, habitat suitability, and climatic conditions affecting carp spawning success are identified. This underscores the need for conserving these ecosystems and implementing effective management strategies to sustain fish populations, especially carp species. By elucidating relationships between hydrological, environmental, and biological parameters, the research enhances our knowledge of aquatic ecosystem dynamics and emphasizes the importance of informed conservation practices. The findings provide insights for future conservation efforts and responsible aquatic resource management. However, further research is recommended to explore the biodiversity of fish, plankton, and other nonpiscine consumers, as well as catchability, for a more comprehensive ecological understanding. Overall, the study highlights the importance of preserving the ecological balance of these habitats to support fish populations and their ecosystems.

## Figures and Tables

**Table 1:** Design of the study site for spawn collection and sampling using Savar net.

Station	Transects	Latitude	Longitude
Surma River	Hajipur (*T*_1_) [32]	24.86532	91.93271
	Muradpur bazar (*T*_*c*_)	24.86444	91.97264
	Vurvuri dor (*T*_2_)	24.87143	92.02049
	Chankhair (*T*_3_)	24.85399	91.93914

Tanguar Haor	Alam duar (*T*_2_) [31]	25.14000	91.08832
	Gulabari- Joypur (*T*_*c*_)	25.14007	91.08734
	Beshkhali, Gulduba (*T*_1_)	25.1418	91.08693
	Rowa beel (*T*_3_)	25.12327	91.08103

**Table 2:** Mean values of water quality parameters at various sampling stations analyzed by using the Kruskal–Wallis test throughout the study duration.

Parameters	Reference value^*∗∗*^ [38–41]	Station		
		Surma River (means ± standard deviation)	Tanguar Haor (means ± standard deviation)	*p* value
Temperature (°C)	20–30	29.73 ± 0.20^a^	29.73 ± 0.20^a^	0.722
DO (ppm)	4–6	5.80 ± 0.12^a^	5.90 ± 0.11^a^	0.183
TDS (ppm)	<400	40.05 ± 0.77^a^	38.81 ± 0.60^a^	0.331
Turbidity (NTU)	—	21.55 ± 0.67^a^	20.71 ± 0.29^a^	0.470
Conductivity (*μ*S/cm)	800–1000	85 ± 0.50^a^	85 ± 0.90^a^	0.191
pH	6.5–8.5	6.80 ± 0.29^a^	6.97 ± 0.31^b^	0.006
Ammonia (ppm)	—	0.18 ± 0.04^a^	0.18 ± 0.02^a^	0.530

^*∗*^Different superscripts indicated significant differences in Kruskal–Wallis test (*p*<0.05). ^*∗∗*^Optimum range of water quality parameters from previous studies.

**Table 3:** (a) The general status of plankton with their diverse groupings was documented from the Surma River. (b) The general status of plankton with their diverse groupings was documented from the Tanguar Haor.

(a) Surma river		
Plankton	Class	Taxa

Phytoplankton	Bacillariophyceae	*Cyclotella*
	Cyanophyaceae	*Spirulina*
		*Oscillatoria*
	Euglenophyceae	—
	Chlorophyceae	*Ankistrodesmus*
		*Spirogyra*
Zooplankton	Rotifer	*Asplanchna*
		*Brachionus*
		*Keratella*
		*Filinia*
	Cladocera	*Sida*
		*Moina*
		*Daphnia*
		*Diaphanosoma*
		*Bosmina*
	Copepods	*Cyclops*
		*Diaptomus*
		*Nauplius*
	Protozoans	—

(b) Tanguar Haor		
Plankton	Class	Taxa

Phytoplankton	Bacillariophyceae	*Navicula*
		*Cymbella*
		*Cyclotella*
		*Synedra*
	Cyanophyceae	*Gloecapsa*
		*Microcystis*
		*Spirulina*
		*Oscillatoria*
		*Anabaena*
		*Gomphosphaeria*
	Euglenophyceae	*Euglena*
		*Phacus*
	Chlorophyceae	*Ankistrodesmus*
		*Chlorella*
		*Closterium*
		*Microspora*
		*Pediastrum*
		*Scenedesmus*
		*Spirogyra*
		*Staurastrum*
		*Tetraedron*
		*Ulothrix*
		*Ceratium*
Zooplankton	Rotifer	*Asplanchna*
		*Brachionus*
		*Keratella*
		*Filinia*
	Cladocera	*Sida*
		*Moina*
		*Daphnia*
		*Diaphanosoma*
		*Bosmina*
	Copepods	*Cyclops*
		*Diaptomus*
		*Nauplius*
	Protozoans	*Difflugia*
		*Arcella*

**Table 4:** Available fish species as recorded from the collected spawn of the Surma River and Tanguar Haor.

Common group	Order	Family	Scientific name	Local name
Carps	Cypriniformes	Cyprinidae	*Labeo calbasu*	Kalibaus
			*Labeo bata*	Bata
			*Labeo gonius*	Gonia

Barbs & minnows	Cypriniformes	Cyprinidae	*Puntius ticto*	Tit puti
			*Puntius sophore*	Jatputi
			*Amblypharyngodon mola*	Mola
			*Osteo brama cotio*	Dhela
			*Esomus danricus*	Darkina

Loaches	Cypriniformes	Cobitidae	*Lepidocephalichthys*	Gutum
			*Guntea*	
			*Botia dario*	Rani

Clupeids	Clupeiformes	Clupeidae	*Gudusia chapra*	Chapila
			*Corica soborna*	Kachki

Snakeheads	Perciformes	Chanidae	*Channa marulius*	Gozar
			*Channa striatas*	Shol
			*Channa punctatus*	Taki
		Nandidae	*Nandus nandus*	Veda, Meni

Perches	Perciformes	Anabantidae	*Anabas testudineus*	Koi
		Cichlidae	*Oreochromis mossambicus*	Tilapia
		Osphronemidae	*Colisa fasciata*	Barokholisha
		Gobiidae	*Glossogobius giuris*	Bele
		Ambassidae	*Parambassis lala*	Lalchanda
			*Pseudambassis baculis*	Chanda
	Beloniformes	Belonidae	*Xenentodon cancila*	Kankila

Catfishes	Siluriformes	Heteropneustidae	*Heteropneustes fossilis*	Shing
		Clariidae	*Clarias batrachus*	Magur
		Schilbeidae	*Ailia coila*	Kajuli
			*Eutropiichthys vacha*	Vacha
		Bagridae	*Sperata aor*	Air
			*Mystus tengara*	Gulshatenga
		Siluridae	*Mystus vittatus*	Tengra
			*Wallago attu*	Boal
			*Ompok pabda*	Pabda

Eels & mud eels	Synbranchiformes	Mastacembelidae	*Macrognathus aculeatus*	Tara baim
			*Mastacembelus pancalus*	Guchibaim
			*Mastacembelus armatus*	Baim
		Synbranchidae	*Monopterus cuchia*	Kuchia

Featherbacks	Osteoglossiformes	Notopteridae	*Chitala chitala*	Chitol

Miscellaneous	Decapoda	Palaemonidae	*Macrobrachium rosenbergii*	Galda
			*Macrobrachium rude*	Gura chinghri

## Data Availability

Raw data used to support the current findings will be available by contacting the corresponding author for reasonable requests.

## Supplementary Materials

Supplementary Materials Supplementary Table 1: Homogeneity test of data for ANOVA comparison. Supplementary Table 2: Kruskal-Wallis ANOVA test.
