# Supplementary material for: Ecological Insights, and Fin Fish Diversity in Carps Spawning Grounds: Case Studies from the Surma River and Tanguar Haor, Bangladesh
Source: Scientifica (Cairo). 2024 Mar 25;2024:7195596. doi: 10.1155/2024/7195596 (PMC10985277; doi:10.1155/2024/7195596)

Supplementary Table 1: Homogeneity test of data for ANOVA comparison.

|  | | | | | | |
| --- | --- | --- | --- | --- | --- | --- |
|  | Kolmogorov-Smirnov^a^ | | | Shapiro-Wilk | | |
|  | Statistic | df | Sig. | Statistic | df | Sig. |
| Tem | .149 | 96 | .000 | .934 | 96 | .000 |
| DO | .058 | 96 | .200^*^ | .991 | 96 | .748 |
| TDS | .103 | 96 | .014 | .968 | 96 | .020 |
| Tur | .217 | 96 | .000 | .528 | 96 | .000 |
| Cond | .172 | 96 | .000 | .640 | 96 | .000 |
| pH | .082 | 96 | .113 | .987 | 96 | .475 |
| Amm | .201 | 96 | .000 | .905 | 96 | .000 |

Supplementary Table 2: Kruskal-Wallis ANOVA test.


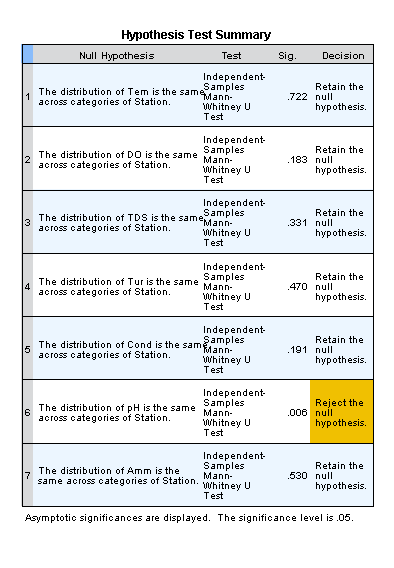


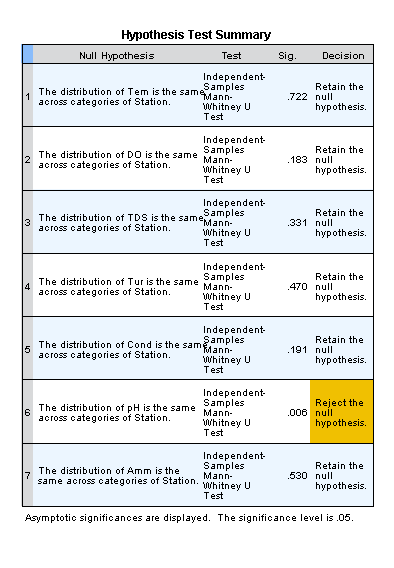

Supplement: Supplementary Materials — Supplementary Table 1: Homogeneity test of data for ANOVA comparison. Supplementary Table 2: Kruskal-Wallis ANOVA test. [file 7195596.f1.docx]
